# Editorial: Motor coordination, cognitive functions, and emotions across the lifespan

**DOI:** 10.3389/fpsyg.2023.1258231

**Published:** 2023-08-04

**Authors:** Valerio Giustino, Daniela Smirni, Simona Pajaujiene, Antonella Muscella, Antonino Bianco

**Affiliations:** ^1^Sport and Exercise Sciences Research Unit, Department of Psychology, Educational Science and Human Movement, University of Palermo, Palermo, Italy; ^2^Department of Psychology, Educational Science and Human Movement, University of Palermo, Palermo, Italy; ^3^Department of Coaching Science, Lithuanian Sports University, Kaunas, Lithuania; ^4^Department of Biological and Environmental Science and Technologies, University of Salento, Lecce, Italy

**Keywords:** motor coordination, motor competence, motor skills, motor performance, children, older people, elderly, athletes

## 1. Introduction

Motor coordination (MC), at every stage of life, represents a Research Topic widely investigated by several research groups since it is related to the quality and the ability level of motor skill competence and is also associated with several physical, cognitive, and emotional domains (Lubans et al., 2010; D'Hondt et al., 2011, 2013; Patti et al., 2017; Battaglia et al., 2020, 2021).

The Research Topic “*Motor coordination, cognitive functions, and emotions across the lifespan*” consists of seven articles (of which five are original articles, one is a study protocol, and one a systematic review with meta-analysis) and covers a variety of aspects of MC across the lifespan (i.e., from preschool children and adolescents to elderly people).

## 2. Original articles (*n* = 5)

The study by Han et al. aimed to explore the association between early fundamental motor skills (FMS), measured through the Test of Gross Motor Development-2 (TGMD-2), and executive function (EF), evaluated using the NIH Toolbox Cognition Battery (NTCB), in a sample of 394 preschool Chinese children recruited from both urban and rural areas. The authors observed that the total score of FMS was significantly and positively correlated with the total score of EF. Moreover, the total score of FMS was a significant predictor of the total score of EF. As for the subtests, the locomotor skill was a significant predictor of inhibition control, working memory, and cognitive flexibility, while the object control skill was a significant predictor of inhibition control. The authors concluded that these findings are of interest to early childhood policymakers, preschool teachers, and researchers for implementing appropriate motor programs integrating cognitive demands in order to improve both motor and cognitive skills in preschool children.

The original article by Esen et al. investigated the impact of a coordination-based movement education model on balance development in 5-year-old children. Specifically, the sample consisted of 42 preschool children, of which 20 were allocated to the experimental group and 22 to the control group. The participants' balance in anterior, posterolateral, and posteromedial directions was measured through the Y Balance Test, using the three-part Y Balance Test platform. The results showed that balance between the experimental and control groups did not differ significantly compared to the pre-test. However, there was a significant difference in all direction scores in favor of the post-test and experimental group compared to the control group and the pre-test. Moreover, although not significant, females achieved higher scores than males. Based on these results, the authors suggest that the coordination-based movement education model had a positive effect on balance in preschoolers.

The goal of the research by Knaier et al. was to investigate secular trends of motor performance (MP) in a sample of 1,183 Swiss children and adolescents, aged 7–18 years, and included from eight studies over a period of 35 years (i.e., from 1983 to 2018). The same motor test [i.e., the Zurich Neuromotor Assessment (ZNA)] was used for this study, which allows the evaluation of five motor components: pure motor, fine motor, dynamic balance, static balance, and contralateral associated movements. The effects of the year of birth and body mass index (BMI) on MP were considered. The authors found that MP remained relatively stable across generations and no secular trends were found in four motor components with the exception of dynamic balance, in which a secular trend was detected independent of the secular trend in BMI.

The aim of the original research by Ma and Luo was to investigate any relationship between physical activity (PA; measured using a triaxial accelerometer), FMS (measured using TGMD-2), and BMI (assessed through anthropometry measurements) in 366 preschoolers of both genders and between 3 and 6 years of age. Another purpose of the study was to explore the differences in PA and FMS between weight status categories (i.e., normal weight and overweight/obese). The main results showed that PA was significantly and positively correlated with both locomotor and object control skills. No correlation between FMS, PA, and BMI was found. Furthermore, no significant differences in FMS between normal weight and overweight/obese children were detected. Normal weight girls reported significantly longer durations of moderate physical activity (MPA) and moderate to vigorous physical activity (MVPA) as well as significantly shorter sedentary periods than overweight/obese girls. As for differences in age and weight status categories, older children showed significantly higher scores in both locomotor and object control skills, and boys spent more time on MVPA and less time on sedentary activity than girls. The authors concluded that PA is positively correlated with FMS and that BMI is not correlated with FMS or PA in preschoolers.

The original article by Slimi et al. aimed to study the effect of adapted basketball sessions on empathic capacity in overweight adolescent girls. The intervention program lasted 7 weeks; each week, participants carried out two adapted basketball teaching-learning sessions, lasting 50 min each. The results were compared with a control group who received an intervention without adaptation (classic basketball sessions). Forty-two participants, aged between 15 and 17 years, were randomly assigned to the experimental group or the control group. Empathy, assessed using the Favre CEC before and after the 7-week program, revealed that the adapted intervention was associated with a significant emotional contagion decrease and splitting of emotions, with an empathy increase observed. No significant difference was found in the control group. These findings demonstrated that adapted basketball sessions could be an effective strategy to improve empathy in overweight girls.

## 3. Study protocol (*n* = 1)

The study protocol by Zhang et al. will assess the effects of Tai Chi practice on insomnia treatment in elderly people with chronic, non-specific lower back pain. Using a randomized, controlled, open-label study design, the research team will recruit a sample of 106 participants who will be randomized into a Tai Chi group that will perform an 8-week Tai Chi program, and a control group that will have an 8-week waiting period, followed by an 8-week Tai Chi program. The main outcomes will be changes in sleep quality and pain intensity. Secondary outcomes will include changes in pain quality, range of motion, physical performance, social support, and overall quality of life.

## 4. Systematic review with meta-analysis (*n* = 1)

The systematic review and meta-analysis by Silva et al. followed the PRISMA 2020 and Cochrane guidelines, evaluating the differences in visual search behavior between expert and novice team sports athletes. The PECOS framework was followed to set the eligibility criteria. Participants were included if they were team sport athletes, healthy, engaged in regular practice, and of any gender or competitive level. As for the exposure, *in situ* or film-based match/game scenarios in which visual search behavior was assessed through eye-tracking technology were included. For comparison, the authors considered at least one group with an expert level to be different from the main group. Regarding outcomes, the studies in this systematic review had to include at least one of the outcomes considered. The authors considered only research published in peer-reviewed journals, with no date or language restrictions. The databases used were EBSCO, PubMed, Scopus, SPORTDiscus, and Web of Science. The risk of bias was determined using the RoBANS tool. From a total of 6,257 records, 22 studies were included. The authors concluded that distinguishing expert team sports athletes from novices in the variables considered was not certain, probably due to the heterogeneity of the included studies.

## 5. Conclusion

In summary, this Research Topic for Frontiers in Psychology presents interesting current studies in the research field of MC, emphasizing the importance of continuing to investigate and add new knowledge on this area to the literature, with further works aiming to develop effective MC programs.

## Author contributions

VG: Writing—original draft. DS: Writing—review and editing. SP: Writing—review and editing. AM: Writing—review and editing. AB: Writing—review and editing.
